# 2405. Extended Incubation of Blood Cultures in the Identification of *Cutibacterium acnes* as the Causative Pathogen in Infective Endocarditis

**DOI:** 10.1093/ofid/ofad500.2025

**Published:** 2023-11-27

**Authors:** Sofia Molina Garcia, Nabin K Shrestha, Steven M Gordon

**Affiliations:** Cleveland Clinic, Cleveland, Ohio; Cleveland Clinic Foundation, Cleveland, OH; Cleveland Clinic Foundation, Cleveland, OH

## Abstract

**Background:**

*Cutibacterium acnes* is an uncommon cause of infective endocarditis (IE). Blood cultures are sometimes held for extended incubation (beyond the standard 5 days) with an expectation that such incubation might allow *C. acnes* to grow. Extended incubation of blood cultures consumes resources, and a justification of such extension requires a demonstration that it increases the likelihood of identifying a causative pathogen.

The purpose of this study was to examine the association between extended incubation of blood cultures and the identification of *C acnes* as the causative pathogen in patients with IE possibly caused by *C. acnes*.

**Methods:**

This was a retrospective case-control study. Episodes of surgically treated IE among patients admitted from July 1, 2007, through Jan 1, 2022 were identified from the Cleveland Clinic Infective Endocarditis Registry, were screened for inclusion in the study. Patients with *C. acnes* IE were case subjects. Controls were selected from patients without an identified pathogen, by propensity score matching on age, gender, admission year, and IE category (native valve endocarditis or prosthetic valve endocarditis), using the nearest neighbor method, from among episodes of IE for whom a causative pathogen was not identified. Extended incubation was defined as incubation of blood cultures for longer than 5 days. The association between extended incubation of blood cultures and *C. acnes* identification was examined using logistic regression.

**Results:**

Thirty-five subjects with *C. acnes* IE were matched to 35 control subjects. Nine of 35 subjects in the *C. acnes* group had at least 1 blood culture held for >5 days compared to only 1 of 35 controls. Five (50%) of those held for extended incubation grew *C. acnes*. Extended incubation was associated with a significantly higher odds of identification of *C. acnes* as the cause of IE (OR 11.8, 95% C.I. 2.0 – 224.1, p-value 0.02).

**Conclusion:**

Among patients with possible *C. acnes* IE, extended incubation of blood cultures is associated with 12-fold higher odds of identifying *C. acnes*.

**Disclosures:**

**All Authors**: No reported disclosures

